# Development of a quantitative methylation-specific droplet digital PCR assay for detecting Dickkopf-related protein 3

**DOI:** 10.1186/s13104-022-06056-6

**Published:** 2022-05-13

**Authors:** Kenji Araki, Ai Kurosawa, Hiromi Kumon

**Affiliations:** 1 Innovation Center Okayama for Nanobio-Targeted Therapy, Okayama University, Okayama, Japan; 2 Watarase Research Center, Kyorin Pharmaceutical Co., Ltd., 1848, Nogi, Nogi-machi, Shimotsuga-gun, Tochigi, 329-0114 Japan; 3 Niimi University, Niimi, Okayama Japan

**Keywords:** Dickkopf-related protein 3, Methylation, Liquid biopsy, Droplet digital polymerase chain reaction

## Abstract

**Objective:**

The detection and monitoring of DNA methylation status in circulating tumor cell DNA (ctDNA) provides critical insights into cancer diagnosis and progression. The methylation status of the Dickkopf-related protein 3 (*DKK3*) promoter region is correlated with the metastasis and recurrence of multiple cancers. Thus, detecting the methylation status via non-invasive methods is essential for the diagnosis and prognosis of cancers. Using a droplet digital polymerase chain reaction approach, we have developed a highly sensitive and quantitative measurement of methylated and unmethylated DKK3 derived from circulating cell-free DNA (ccfDNA).

**Results:**

We confirmed the specificity of droplet digital methylation specific polymerase chain reaction (ddMSP). We selected the optimal bisulfite conversion method using commercially available kits. We validated the ddMSP analysis system by analyzing the methylation status of genomic DNA extracted from cultured mesothelioma cells and mesothelial cells. Our system quantified approximately 30 copies of cell-free DNA per 4 mL, which is sufficient for detecting ctDNA. Finally, we quantified methylated and unmethylated DKK3 copies in ccfDNA from 21 patients with malignant mesothelioma.

**Supplementary Information:**

The online version contains supplementary material available at 10.1186/s13104-022-06056-6.

## Introduction

Circulating cell-free DNA (ccfDNA) is released into the blood flow from cells that undergo apoptosis and necrosis, and is released actively from various cells. ccfDNA typically exists as double-stranded fragments of approximately 150–200 base pairs per unit size of the nucleosome. Based on this, circulating tumor DNA (ctDNA) molecules reflect the genome or epigenome of tumor tissues. However, the proportion of ctDNA in total ccfDNA is highly inconsistent, ranging from < 0.1 to 10% [1]. Therefore, sensitive assays that can quantify ctDNA in ccfDNA are sought after.

Dickkopf-related protein 3 (*DKK3*) belongs to a family of human *DKK*-related genes, including *DKK1*, *DKK2*, *DKK3*, and *DKK4*, which act as secretory Wnt signaling modulators [2]. *DKK3* expression is epigenetically silenced via methylation of CpG islands in promoters in most human cancer cells [3]. The loss of *DKK3* expression activates Wnt signaling to contribute to metastatic cancer and recurrent tumor development in gastric [4] and breast cancers [5]. To date, several methods have been developed for the detection of *DKK3* methylation status in formalin-fixed paraffin-embedded (FFPE) specimens [6]. However, the analysis of FFPE specimens is invasive and unsuitable for routine clinical testing. We modified a previously reported method [7] to determine the methylation status of the *DKK3* promoter region in ctDNA. Although methylation-specific polymerase chain reaction (PCR) assays can measure ultra-low amounts of samples, they are limited by various factors, such as few genetic loci evaluated for methylation and non-methylation-specific amplification due to complicated primer and probe design. ddPCR can measure the methylation of specific genes using sera from mesothelioma patients [8], indicating that ddPCR is a suitable system for measuring trace amounts of circulating DNA in terms of sensitivity. In this study, we developed a novel method for evaluating the methylation status of the *DKK3* promoter via droplet digital PCR (ddPCR) detection of ccfDNA.

## Main text

### Material and methods

#### Purification of ccfDNA from human serum or plasma samples

Unless otherwise stated, 0.5 mL (Eppendorf, Tokyo, Japan) or 2.0 mL (Eppendorf) Protein LoBind tubes were used. The QIAamp MinElute ccfDNA Mini Kit (Qiagen, Tokyo, Japan) was used to extract ccfDNA from 4 mL of serum, plasma, or culture supernatant, according to the manufacturer’s instructions. The concentration of ccfDNA was measured using the Qubit dsDNA HS Assay Kit (Thermo Fisher Scientific, Yokohama, Japan).

#### Bisulfite conversion of ccfDNA as a template for droplet digital methylation-specific PCR (ddMSP)

Bisulfite conversion was performed using the EZ DNA Methylation-Lightning Kit (Zymo Research, Orange, CA, USA), Premium Bisulfite Kit (Diagenode Diagnostics, Liège, Belgium), and EpiJET Bisulfite Conversion Kit (Thermo Fisher Scientific) according to the manufacturer’s instructions. The concentration of bisulfite-treated ccfDNA was measured using the Qubit ssDNA Assay Kit (Thermo Fisher Scientific).

#### ddMSP

**Fig. 1:**
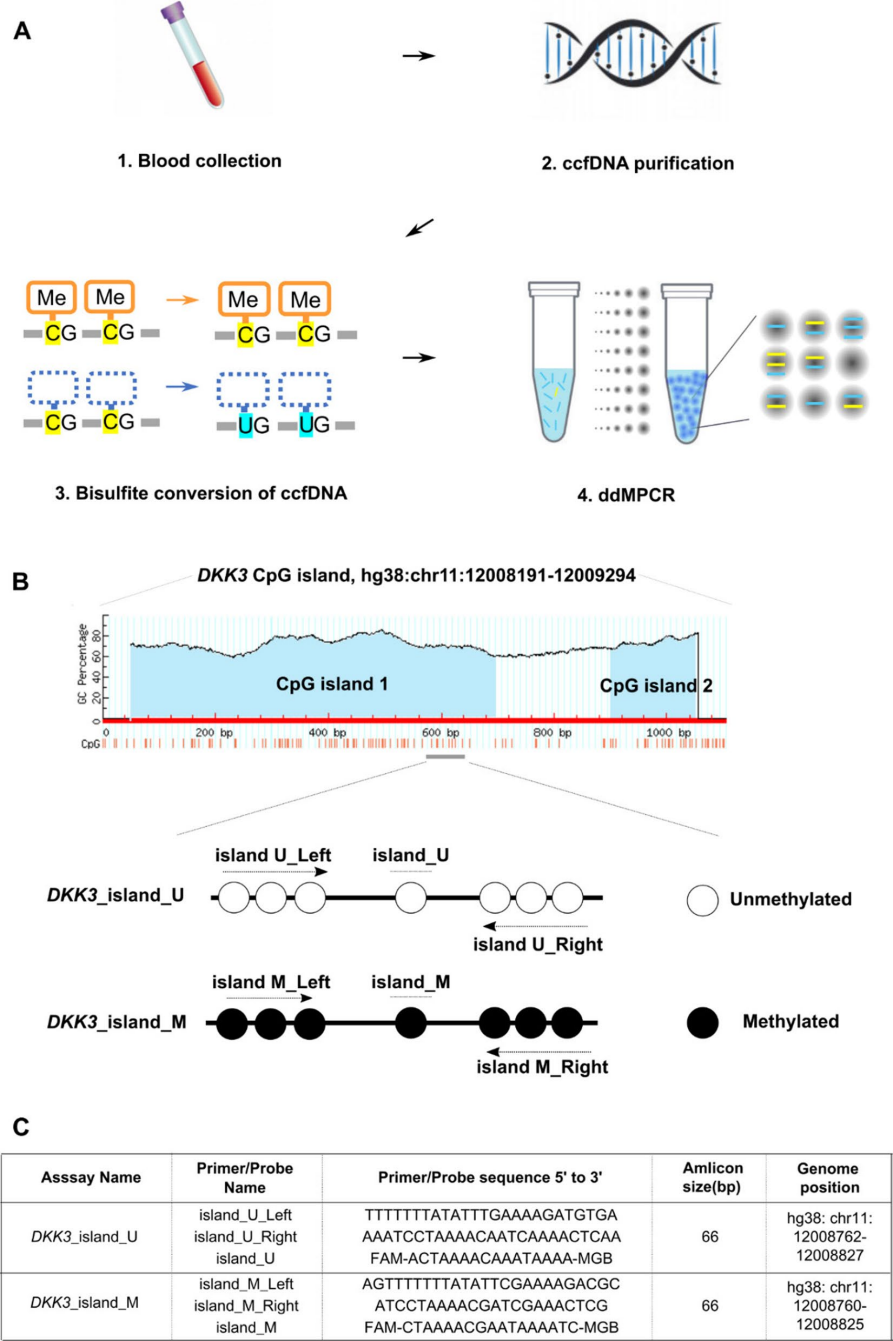
Rational primer and probe design for ddMSP. **A** Graphical abstract of the detection system. **B** The prediction of CpG islands was performed using MethPrimer software. Cytosine bases that can be methylated are indicated in red, and the CpG island is presented in the blue area. **C** Sequences of the primer pairs and probes used in each assay, amplicon size, and the genome position. *ccfDNA* circulating cell-free DNA; *ddMPCR* droplet digital methylation-specific polymerase chain reaction

The sequence of the *DKK3* CpG island (position: chr11:12008191–12009294, band: 11p15.3, and genomic size: 1,104 bp), was downloaded from the UCSC genome browser (http://genome.ucsc.edu/) and analyzed using MethPrimer 2.0 (http://www.urogene.org/methprimer2/tester-invitation.html) [9]. The primers and Taqman-MGB probes were designed in our laboratory and synthesized by Thermo Fisher Scientific. Unmethylated *DKK3*-derived sequences were amplified using the *DKK3*_island_U assay, and methylated *DKK3*-derived sequences were amplified using the *DKK3*_island_M assay (Fig. 1C). ddPCR was performed using the QX200 system (Bio-Rad Laboratories, Hercules, CA, USA). EpiScope-Methylated HCT116 gDNA (Takara Bio Inc., Otsu, Japan) and unmethylated HCT116 DKO gDNA (Takara-Bio) were both subject to bisulfite conversion and used as positive and negative controls for methylation-specific PCR. The total volume of the PCR mixture in the assay was 20 µL, comprising 10 µL of ddPCR Supermix for Probes (No dUTP) (Bio-Rad Laboratories), 0.9 µM of each primer, 0.25 µM of each probe, and 200 µM of dNTP. The following PCR conditions were used for ddPCR: 10 min at 95 °C for DNA polymerase activation, followed by 40 cycles of 30 s at 94 °C for denaturation and 1 min at 50 and 56 °C (*DKK3*_island_U assay and *DKK3*_island_M assay, respectively) for annealing and extension, and termination at 98 °C for 10 min for DNA polymerase deactivation. Methylation- and non-methylation-specific PCR was performed using a C1000 Touch Thermal Cycler with 96 Deep well reaction modules (Bio-Rad Laboratories) in each methylation-specific PCR. The PCR products were read and analyzed using the QX-200 droplet reader (Bio-Rad Laboratories) and QuantaSoft analysis software (Version 1.7.4) (Bio-Rad Laboratories).

#### Clinical samples

Normal human serum (#S1-100ML, Lot_3291313; Millipore, Japan, Tokyo, Japan) and normal human plasma samples (#12271430, Lot_BJ12440A; Tennessee Blood Services Corporation, Memphis, TN, USA) were used as normal controls. Peripheral blood samples were collected from 21 patients with malignant mesothelioma. The details are shown in Additional files 2 and 3. Patients’ serum was pooled, and ccfDNA was purified from 4 mL.

### Results and discussion

#### Summary of the assay developed in this study

A scheme of the novel method developed for detecting the methylation state of *DKK3* via ddMSP using liquid biopsy is presented in Fig. 1A. First, we collected approximately 4 mL of pooled serum samples from patients with malignant mesothelioma. We then extracted ccfDNA using the QIAamp MinElute ccfDNA Mini Kit. The ccfDNA was subjected to bisulfite conversion using the EpiJET Bisulfite Conversion Kit. Finally, we quantified the copy number of methylated and unmethylated regions in the CpG island *DKK3* promoter region via ddMSP using the QX200 system.

#### Primer and probe design for methylated and unmethylated DKK3 promoter regions

The primer and probe designs used for methylation-specific-PCR are crucial, so they were designed in the following flow. We analyzed the CpG islands (position: chr11:12008191–12009294) of the *DKK3* promoter using MethPrimer software and designed methylation-specific primers (Fig. 1B) based on four considerations: (i) multiple CpG sites were included in the target amplicon to increase the selectivity; (ii) the size was as small as possible to increase the sensitivity of ccfDNA detection; (iii) the frequency of single nucleotide polymorphisms (SNPs) was relatively low in the target sequences; (iv) the nearly identical region of the genome was used for PCR in both cases of methylation and methylation alleles. Consequently, we selected one potential PCR amplification region for ddMSP including seven CpG sites (Fig. 1B). Furthermore, the methylation of cytosine immediately adjacent to these target sequences was observed among ten types of cancers with significant differences [10] (Additional file 1). This amplification region had no common SNPs based on Short Genetic Variants from dbSNP release 153 (https://www.ncbi.nlm.nih.gov/snp/). Therefore, we designed primers and TaqMan probe pairs based on these observations (Fig. 1C).

#### Specificity of the ddMSP assay

**Fig. 2:**
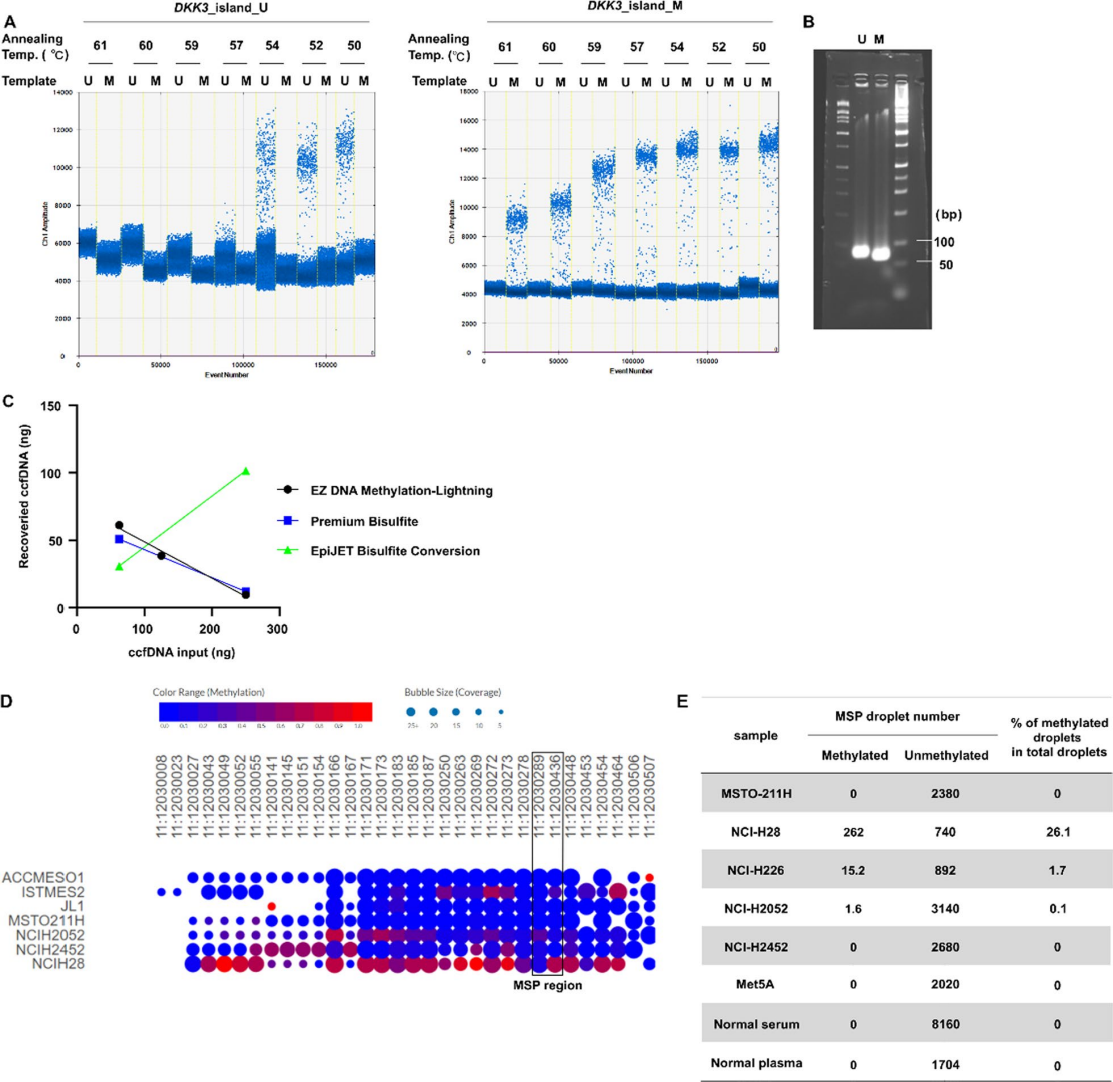
Confirmation of specificity for methylated and unmethylated templates in ddPCR and validation in mesothelioma cell line. **A** Gradient PCR was performed within an annealing temperature range of 50–61 °C with the matched or mismatched template. *U* bisulfited-fully unmethylated genome and *M* bisulfited-fully methylated genome. **B** Each PCR product was subjected to electrophoresis. **C** Changes in the amount of ccfDNA after bisulfite treatment using each kit. Selection of bisulfite conversion kit for ccfDNA. The X-axis shows the ccfDNA input (the amount of DNA before bisulfite conversion), and Y-axis shows the number of recovered ccfDNA (the amount of DNA after bisulfite conversion). The experiments were performed in duplicate, and each mean value was plotted and fitted into a linear regression model. The copy number of methylated *DKK3* in mesothelioma cell lines, mesothelial cells, and non-cancer ccfDNA was determined. **D** The methylation status of *DKK3* is shown based on analysis of reduced representation bisulfite sequencing (https://depmap.org/portal/). **E** Copy number of methylated and unmethylated *DKK3* in human mesothelioma cell lines (MSTO-211H, NCI-H28, NCI-H226, and NCI-H2052), mesothelial cell line (Met5A), and non-cancer cell-free DNA (derived from 2 donors). *ddPCR* droplet digital polymerase chain reaction; *ccfDNA* circulating cell-free DNA; *DKK3* Dickkopf-related protein

Since the sequences of amplicon for methylation and unmethylation are very similar, it is important to confirm the specificity. Confirming the specificity of ddMSP is very important for detecting a small percentage of existence of ctDNA. As a validation study, we first determined the specificity of each ddMSP reaction using matched and mismatched templates with optimization of the annealing temperature. We confirmed the selectivity of each PCR in the *DKK3*_island_U and *DKK3*_island_M assays (Fig. 2A). Non-specific reactions were not observed in reaction conditions using the mismatched template. We also optimized the annealing temperature for each PCR. The optimized temperatures were 56 and 50 °C for methylated and unmethylated PCRs, respectively. In addition, we confirmed the specificity of the amplicon size via electrophoresis (Fig. 2B), and the amplicon sequence was verified (data not shown).

#### Comparison of three commercial bisulfite conversion kits for converting ccfDNA derived from ccfDNA

The bisulfite treatment causes further fragmentation of the DNA, which is expected to have a particular impact on the detection system in the case of ccfDNA. Therefore, it is important to perform ddPCR after appropriate bisulfite treatment. We compared three major commercial bisulfite conversion kits to evaluate the recovery rate in determination of copies of unmethylated *DKK3* derived from ccfDNA (Fig. 2C). An undiluted solution of ccfDNA extracted from serum and a sample diluted two or four times with nuclease-free water was subjected to bisulfite conversion. The EZ DNA Methylation-Lightning Kit and Premium Bisulfite Kit showed a negative correlation between the copy numbers of ccfDNA before bisulfite conversion and after bisulfite conversion, implying that impurities after bisulfite treatment may affect the detection system. In contrast, the EpiJET Bisulfite Conversion kit showed a positive correlation with the recovery rate (approximately 50%) and the copies of input. Although the reason for this finding is not known, it was established that the assay using EpiJET bisulfite transformation does not significantly impact our detection system. Consequently, we selected the EpiJET Bisulfite Conversion kit for use in the ddMSP system.

#### Measurement of ctDNA in malignant mesothelioma cell lines and mesothelial cells

To validate the ddMSP analysis system, we analyzed the methylation status of genomic DNA extracted from cultured mesothelioma cells (MSTO-211H, NCI-H28, NCI-H226, NCI-H2052, and NCI-H2452) and mesothelial cells (Met5A). Sources of cells and culture methods are shown in Additional file 2. The results of reduced bisulfite sequencing against *DKK3* CpG island (hg19: chr11: 12030008–12030507) were downloaded and compared as a reference (Fig. 2D). Methylated *DKK3* in genomic DNA copies was detected in NCI-H28 and NCI-H226 cells, and in NCI-H2052 cells to a lesser extent (Fig. 2E). Methylated *DKK3* sequences were not detected in MSTO-211H and NCI-H2452 cells. In comparison with the cancer cell line encyclopedia database, the trend of methylation rate was very similar to the results of this experiment. No copies of the methylated genome were detected in the assay using the normal mesothelial cell line Met5A. Furthermore, when ccfDNA derived from normal serum and normal plasma (collected from different individuals) was examined, no copies of the methylated genome were detected. These results suggested that these MSP regions were specific to the tumor tissue.

#### Detection and quantification limits of the ddMSP assay

**Fig. 3:**
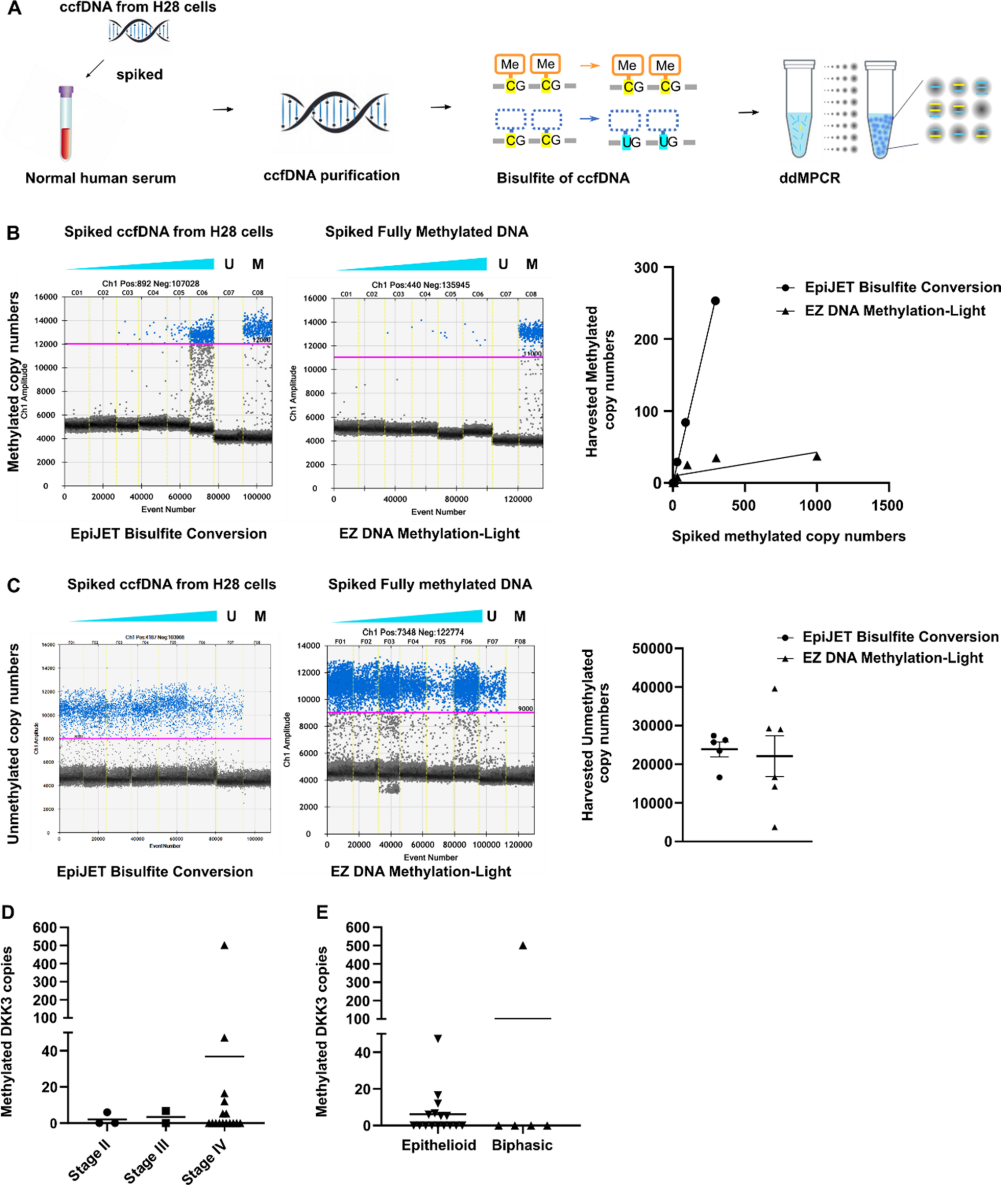
Spike and recovery test of the ddMSP detection system and validation using samples of patients with mesothelioma. **A** Human serum was spiked with pre-determined copies of ccfDNA from H28 mesothelioma cells or fully methylated genomic DNA, and ccfDNA was purified following bisulfite conversion. The copy numbers of methylated *DKK3* were quantified via ddPCR. **B** Typical scatter plots obtained from the experiments are shown. The X-axis shows the number of copies spiked into human serum; the Y-axis shows the number of copies recovered after the process. From each point, a linear regression curve was drawn in each bisulfite conversion. **C** Typical scatter plots obtained from the experiments. The Y-axis shows the percentage of methylated *DKK3* copies per unmethylated *DKK3* derived from human serum. Assays were performed in two independent experiments, and the representative one is shown. Determination of the methylation status in the *DKK3* promoter region of ccfDNA in patients with malignant mesothelioma. **D** Patients with mesothelioma were categorized based on stage; the y-axis shows the number of methylated copies per 4 mL of serum. **E** Patients with mesothelioma were classified based on disease type, and the number of methylated copies for each disease type is shown on the Y-axis. The mean value for each population is indicated as a bar. *ddMSP* droplet digital methylation-specific polymerase chain reaction; *ccfDNA* circulating cell-free DNA; *DKK3* Dickkopf-related protein

A low detection limit is critical for the detection of ctDNA in liquid biopsies. Therefore, we quantified the lower detection limit of the ddMSP assay. We spiked normal human serum with NCI-H28 cell-free DNA or fully methylated genome, whose copy number was previously quantified using TaqMan Copy Number Reference Assay RNase P (Thermo Fisher Scientific) (Fig. 3A), and determined the recovery rate after the purification of ccfDNA following bisulfite conversion by quantifying the number of copies of fully methylated DNA. We estimated that most cancers show more than 40 copies of ctDNA in 4.0 mL of patient serum [11]. Therefore, if the assay can detect 30 copies of ctDNA in 4.0 mL, the sensitivity is considered sufficient. Our system quantified approximately 30 copies of cell-free DNA per 4 mL, which is sufficient for detecting ctDNA (Fig. 3B). The data were also analyzed to determine whether a certain amount of unmethylated *DKK3* could be recovered. The EpiJET Bisulfite Conversion kit showed a lower coefficient of variation value (0.18) (Fig. 3C) than that of the EZ DNA Methylation-Lightning Kit (0.58). We were able to detect a very small amount (< 0.2%) of methylated DNA, which is considered to be sufficiently sensitive.

#### Determination of DKK3 copies in serum samples from malignant mesothelioma via ddMSP

To verify that clinical samples can be measured using our measurement system, we quantified methylated and unmethylated *DKK3* copies in ccfDNA from 21 patients with malignant mesothelioma. Patient characteristics are shown in Additional file 4. We detected 5–500 copies of methylated *DKK3* in 4 mL of serum samples of eight patients. In contrast, no copies were detected in serum samples of 13 patients. Furthermore, the number of methylated copies tended to increase as the stage progressed, possibly reflecting changes in tumor size or changes in tumor properties (Fig. 3D). Differences based on disease type cannot be discussed because of the small number of cases (Fig. 3E). Based on these results, this assay system can be used to analyze clinical samples.

### Conclusion

Using our novel method, we selectively and quantitatively measured methylated and unmethylated *DKK3* in ccfDNA.

### Limitations

One limitation of this study is that we did not evaluate the concordance rate with local tumor samples; therefore, the extent to which the peripheral blood results reflect the methylation status in the local tumor is not known. In the future, data such as the positive concordance rate with local test results such as FFPE samples should be evaluated*.*

## Supplementary Information

**Additional file 1: Figure S1.** The β-value [methylated/(methylated + unmethylated cytosine)] in cg13259205 is shown across each type of cancer (red) compared with each normal tissue (green). TCGA data were downloaded from http://www.bioinfo-zs.com/smartapp/, and box plots were generated. *ACC* adrenocortical carcinoma; *BLCA* bladder urothelial carcinoma; *BRCA* breast invasive carcinoma; *CESC* cervical squamous cell carcinoma and endocervical adenocarcinoma; *CHOL* cholangiocarcinoma; *COAD* colon adenocarcinoma; *DLBC* diffuse large B-cell lymphoma; *ESCA* esophageal carcinoma; *GBM* glioblastoma multiforme; *HNSC* head and neck squamous cell carcinoma; *KICH* kidney chromophobe; *KIRC* kidney renal clear cell carcinoma; *KIRP* kidney renal papillary cell carcinoma; *LAML* acute myeloid leukemia; *LGG* brain lower grade glioma; *LIHC* liver hepatocellular carcinoma; *LUAD* lung adenocarcinoma; *LUSC* lung squamous cell carcinoma; *DLBC* lymphoid neoplasm diffuse large B-cell lymphoma; *MESO* mesothelioma; *OV* ovarian serous cystadenocarcinoma; *PAAD* pancreatic adenocarcinoma; *PCPG* pheochromocytoma and paraganglioma; *PRAD* prostate adenocarcinoma; *READ* rectum adenocarcinoma; *SARC* sarcoma; *SKCM* skin cutaneous melanoma; *STAD* stomach adenocarcinoma; *TGCT* testicular germ cell tumors; *THYM* thymoma; *THCA* thyroid carcinoma; *UCS* uterine carcinosarcoma; *UCEC* uterine corpus endometrial carcinoma; *UVM* uveal melanoma; *TCGA* The Cancer Genome Atlas.

**Additional file 2.** Supplemental methods

**Additional file 3: Table S1.** Inclusion criteria and exclusion criteria.

**Additional file 4: Table S2.** Clinical characteristics of patients with malignant mesothelioma (N = 21).

## Data Availability

The datasets used and/or analyzed during the current study are available from the corresponding author on reasonable request.

## Abbreviations

ccfDNA: Circulating cell-free DNA
ctDNA: Circulating tumor DNA
ddPCR: Droplet digital polymerase chain reaction
ddMSP: Methylation-specific ddPCR
FFPE: Formalin-fixed and paraffin-embedded samples
