# Supplementary material for: Development of a quantitative methylation-specific droplet digital PCR assay for detecting Dickkopf-related protein 3
Source: BMC Res Notes. 2022 May 13;15:169. doi: 10.1186/s13104-022-06056-6 (PMC9103039; doi:10.1186/s13104-022-06056-6)
Supplement: Supplementary file 1 — Additional file 1: Figure S1. The β-value [methylated/(methylated + unmethylated cytosine)] in cg13259205 is shown across each type of cancer (red) compared with each normal tissue (green). TCGA data were downloaded from http://www.bioinfo-zs.com/smartapp/, and box plots were generated. ACC adrenocortical carcinoma; BLCA bladder urothelial carcinoma; BRCA breast invasive carcinoma; CESC cervical squamous cell carcinoma and endocervical adenocarcinoma; CHOL cholangiocarcinoma; COAD colon adenocarcinoma; DLBC diffuse large B-cell lymphoma; ESCA esophageal carcinoma; GBM glioblastoma multiforme; HNSC head and neck squamous cell carcinoma; KICH kidney chromophobe; KIRC kidney renal clear cell carcinoma; KIRP kidney renal papillary cell carcinoma; LAML acute myeloid leukemia; LGG brain lower grade glioma; LIHC liver hepatocellular carcinoma; LUAD lung adenocarcinoma; LUSC lung squamous cell carcinoma; DLBC lymphoid neoplasm diffuse large B-cell lymphoma; MESO mesothelioma; OV ovarian serous cystadenocarcinoma; PAAD pancreatic adenocarcinoma; PCPG pheochromocytoma and paraganglioma; PRAD prostate adenocarcinoma; READ rectum adenocarcinoma; SARC sarcoma; SKCM skin cutaneous melanoma; STAD stomach adenocarcinoma; TGCT testicular germ cell tumors; THYM thymoma; THCA thyroid carcinoma; UCS uterine carcinosarcoma; UCEC uterine corpus endometrial carcinoma; UVM uveal melanoma; TCGA The Cancer Genome Atlas. [file 13104_2022_6056_MOESM1_ESM.docx]

Figure S1. The β-value [methylated/(methylated+unmethylated cytosine)] in cg13259205 is shown across each type of cancer (red) compared with each normal tissue (green). TCGA data were downloaded from http://www.bioinfo-zs.com/smartapp/, and box plots were generated. ACC, adrenocortical carcinoma; BLCA, bladder urothelial carcinoma; BRCA, breast invasive carcinoma; CESC, cervical squamous cell carcinoma and endocervical adenocarcinoma; CHOL, cholangiocarcinoma; COAD, colon adenocarcinoma; DLBC, diffuse large B-cell lymphoma; ESCA, esophageal carcinoma; GBM, glioblastoma multiforme; HNSC, head and neck squamous cell carcinoma; KICH, kidney chromophobe; KIRC, kidney renal clear cell carcinoma; KIRP, kidney renal papillary cell carcinoma; LAML, acute myeloid leukemia; LGG, brain lower grade glioma; LIHC, liver hepatocellular carcinoma; LUAD, lung adenocarcinoma; LUSC, lung squamous cell carcinoma; DLBC, lymphoid neoplasm diffuse large B-cell lymphoma; MESO, mesothelioma; OV, ovarian serous cystadenocarcinoma; PAAD, pancreatic adenocarcinoma; PCPG; pheochromocytoma and paraganglioma; PRAD, Prostate adenocarcinoma; READ; rectum adenocarcinoma; SARC, sarcoma; SKCM, skin cutaneous melanoma; STAD, stomach adenocarcinoma; TGCT, testicular germ cell tumors; THYM, thymoma; THCA, thyroid carcinoma; UCS, uterine carcinosarcoma; UCEC, uterine corpus endometrial carcinoma; UVM, uveal melanoma; TCGA, The Cancer Genome Atlas.
