# Supplementary material for: Development of a quantitative methylation-specific droplet digital PCR assay for detecting Dickkopf-related protein 3
Source: BMC Res Notes. 2022 May 13;15:169. doi: 10.1186/s13104-022-06056-6 (PMC9103039; doi:10.1186/s13104-022-06056-6)
Supplement: Supplementary file 2 — Additional file 2. Supplemental methods [file 13104_2022_6056_MOESM2_ESM.docx]

*Clinical samples*

We collected 9 ml peripheral blood samples from 21 cases of malignant mesothelioma at National Cancer Center Hospital (Tokyo, Japan), Hiroshima University Hospital (Hiroshima, Japan), Okayama University Hospital (Okayama, Japan), Ube Medical Center (Yamaguchi, Japan), Okayama Rosai Hospital (Okayama, Japan), Kyushu University Hospital (Fukuoka, Japan), The Hospital of Hyogo College of Medicine (Hyogo, Japan), Chiba University Hospital (Chiba, Japan), Hyogo Prefectural Amagasaki General Medical Center (Hyogo, Japan), and Otemae Hospital (Osaka, Japan). Inclusion criteria and exclusion criteria are described in Table I. The blood samples were taken with a blood collection tube (Terumo Venoject II, Terumo, Tokyo, Japan) and incubated at room temperature for 30 mins, and centrifuged at 1,000 × g for 15 min at 4°C, and the separated serum samples were stored at −80°C at the respective institutions. This study was conducted with the approval of the Institutional Review Board/Ethical Committee of the respective institutions; each of the participants provided written informed consent for the sample collection. All the experiments were performed in accordance with the Declaration of Helsinki.

*Cell culture*

Human malignant mesothelioma cell lines including MSTO-211H, NCI-H28, NCI-H226, NCI-H2052, NCI-H2452, and immortalized normal mesothelial cell line MeT-5A were obtained from the American Type Culture Collection (ATCC, Manassas, VA, USA) and used for validation study for ddPCR detection system. These cell lines were cultured according to the manufacturer’s instructions. Human malignant mesothelioma cell lines were maintained in RPMI-1640 medium (Thermo Fisher Scientific, Yokohama, Japan) supplemented with 10% FBS, and MeT5A were cultured in Medium 199 containing 1.5 g/L sodium bicarbonate, 10% FBS (Biowest, Nuaillé, France), 3.3 nM epidermal growth factor, 400 nM hydrocortisone, 870 nM zinc-free bovine insulin, and 20 mM HEPES in a humidified atmosphere at 37 °C with 5% CO_2_.
