# Supplementary material for: Development of a quantitative methylation-specific droplet digital PCR assay for detecting Dickkopf-related protein 3
Source: BMC Res Notes. 2022 May 13;15:169. doi: 10.1186/s13104-022-06056-6 (PMC9103039; doi:10.1186/s13104-022-06056-6)
Supplement: Supplementary file 3 — Additional file 3: Table S1. Inclusion criteria and exclusion criteria. [file 13104_2022_6056_MOESM3_ESM.docx]

Table I. Inclusion criteria and exclusion criteria

| Inclusion criteria |
| --- |
| - Minimum age is 20 years old - Pathologically confirmed malignant pleural mesothelioma, which is unresectable - Patients with one or more measurable disease - Eastern Cooperative Oncology Group Performance Status < 1 - Patients who have adequate main organs function - Patients who signed written informed consent - Patients who have adequate main organs function |
| Exclusion criteria |
| - Patients with simultaneous tumor and/or metachronous tumor (excluding patients with them whose disease-free interval is over two years) - Active infections - Symptomatic brain metastasis requiring treatment - Pulmonary fibrosis or interstitial pneumonia - Cardiac effusion requiring treatment - Comorbidities requiring systemic corticosteroid or an immunosuppressants |
