# Supplementary material for: Development of a quantitative methylation-specific droplet digital PCR assay for detecting Dickkopf-related protein 3
Source: BMC Res Notes. 2022 May 13;15:169. doi: 10.1186/s13104-022-06056-6 (PMC9103039; doi:10.1186/s13104-022-06056-6)
Supplement: Supplementary file 4 — Additional file 4: Table S2. Clinical characteristics of patients with malignant mesothelioma (N = 21). [file 13104_2022_6056_MOESM4_ESM.docx]

| Table II. Clinical characteristics of patients with malignant mesothelioma (N=21) | |
| --- | --- |
|  |  |
| Characteristics | Value |
|  |  |
| Gender, n (%) |  |
| Male | 20 (95.2 %) |
| Female | 1 (4.8 %) |
| Age, years |  |
| Median | 68.0 (51-85) |
| Histology, n (%) |  |
| Epithelial type | 16 (76.2 %) |
| Biphasic type | 5 (23.8 %) |
| Stages, n (%) |  |
| 2 | 3 (14.3 %) |
| 3 | 2 (9.5%) |
| 4 | 16 (76.2%) |
